# Treatment of Epilepsy

**Published:** 1861-01

**Authors:** 


*Treatment of Epilepsy.*—Dr. Fabre reports seven cases of confirmed and well-marked epilepsy, in which pills of the hydrocyanate of iron have effected cures. He alludes also to numerous cases in which the same substance has been successfully imployed by Dr. Roux, of Brignolles, and adduces the testimony of others in support of the advantageous effect of this preparation, which has been employed since 1829 in chorea and other neuroses complicated with chlorosis.—*Revue de Malgaigne; Journ. Mat. Med.*

Dr. G. S. Bailey, a retired physician of Iowa, states in a letter to the editors of the last-named journal, that his only son, after having been treated six years for epilepsy with every remedy that medical skill could suggest, without success, was finally cured with the hydrocyanate of iron, by Prof. D. L. McGugin, of Keokuk. The formula employed corresponds with the one used by Dr. Treat (*Cin. Lancet and Observer, June*, 1860, p. 383): hydrocyanate of iron, one drachm; powder of valerian, two drachms; extract of Indian hemp, one drachm being originally added by McGugin. Make into one hundred and twenty pills. One of them is to be taken three times a day, gradually increased to four.

Dr. Max Maresk, physician of an establishment for the insane at Vienna, submitted some epileptic patients to the influence of atropine. Out of eight cases taken from the female department, three were completely cured, and the condition of the five others notably ameliorated. Ten other patients, four men and six women, were selected from the department of the incurable insane, for the same experiment. Eight of these experienced a marked diminution in the violence and frequency of their epileptic attacks, as well as in the acerbation of their physical trouble. One-fiftieth of a grain of atropine gave rise, in every case, to the phenomena habitually following the administration of this agent; the patients became habituated to them, although they never ceased during the entire treatment. In every case the pulse lost eight or twelve pulsations during the first hour after taking the remedy, but resumed its normal frequency as soon as the other phenomena manifested themselves. The atropine was administered in a solution of one grain in five hundred drops of rectified alcohol; five or ten drops of this constituting a dose, which is administered once daily' in the morning before breakfast. Coffee, tea and chocolate interfere with the action of the atropine. It is continued for sixty or ninety days, and then resumed after an interval of from thirty to forty-five days. It favors and augments menstruation, and but rarely gives rise to constipation, more frequently to diarrhœa, necessitating, when severe, suspension of its administration for some days. —*L'Union Med.*; *N. O. Med. and Surg. Journ.*

Selinum palustre, in powder, effected a complete cure in the hands of Dr. Th. Herpin, in four cases of idiopathic and partly of inherited epilepsy. He administered from one to four ounces during the week, divided in twenty-four doses, of which three or four were taken daily. According to Dr. Tagod (*Bouchardt's Annuaire de Therap.*) the root and herb of peucedanum austriacum are still more efficacious. He gave two grammes of the powder three times daily.—*North Amer. Med.-Chir. Rev.*, *from Bull. de Therap.*

Mr. E. Baines reports a case with the powdered water-plantain, alisma plantago, four grains twice daily, increased a grain every third day. But the root must be collected at the end of August. This deserves a more extensive trial.—*London Lancet.*

